# MicroRNA-21: A Positive Regulator for Optimal Production of Type I and Type III Interferon by Plasmacytoid Dendritic Cells

**DOI:** 10.3389/fimmu.2017.00947

**Published:** 2017-08-21

**Authors:** Fang Liu, Chunxi Liu, Xiaoyu Hu, Yingli Shang, Li Wu

**Affiliations:** ^1^Institute for Immunology and School of Medicine, Tsinghua University, Beijing, China; ^2^College of Veterinary Medicine, Shandong Agricultural University, Taian, China; ^3^Tsinghua-Peking Center for Life Sciences, Tsinghua University, Beijing, China

**Keywords:** microRNA-21, innate immunity, interferons, toll-like receptors, plasmacytoid dendritic cell, phosphatase and tensin homolog

## Abstract

Plasmacytoid dendritic cells (pDCs) are the major producers of type I and type III interferons (IFNs) that play essential roles in host antiviral immunity. MicroRNAs (miRs) are small, noncoding RNAs that can modulate many immune processes. Although molecular regulation of type I IFN production by pDCs has been studied extensively, the regulation of type III IFN production has not been studied thoroughly, particularly at posttranscriptional level. We show here that miR-21 is an essential positive regulator for the production of both IFN-α and IFN-λ by pDCs and for promoting host defense against viral infection. miR-21 was markedly upregulated in toll-like receptor (TLR)-activated pDCs and was crucial for TLR7/9 ligand- or herpesvirus-induced production of IFN-α and IFN-λ by pDCs. miR-21-deficient pDCs produced significantly lower levels of IFN-α and IFN-λ on activation than those by wild-type pDCs. Impaired antiviral immune responses were also observed in miR-21-deficient mice. Mechanistically, we identified phosphatase and tensin homolog (PTEN) as the major target of miR-21 in pDCs, and miR-21 deficiency resulted in increased expression of PTEN that suppressed TLR-mediated activation of PI3K-Akt-mTOR signaling in pDCs. Hence, our findings provide evidence that miR-21 positively regulates both IFN-α and IFN-λ production and identify an important role for miR-21 in regulating the function of pDCs and in host antiviral immunity.

## Introduction

Interferons (IFNs) are key mediators in innate and adaptive immune responses against viral infection. It has been well established that macrophages, dendritic cells (DCs), fibroblasts and epithelial cells are capable of producing IFNs in response to virus infections *in vivo* (1–3). Among these cells, plasmacytoid dendritic cells (pDCs) are a unique cell subset that specializes in the production of large amounts of type I IFNs (4). pDCs can also secrete other inflammatory cytokines such as interleukin-6 (IL-6) and tumor necrosis factor alpha (TNF-α). In addition, activation of pDCs can lead to production of type III IFNs, also termed IFN-λs (IFN-λ1/2/3) or interleukin 29 and interleukin 28A/B. Type III IFNs are the most recently described antiviral cytokines that are evolutionarily distant to the classical antiviral type I IFNs but exhibit similar antiviral activities to type I IFNs (5). Both type I and III IFNs are induced in response to diverse viruses and toll-like receptor (TLR) agonists (6). Recognition of viruses by pDCs is mainly mediated by TLRs such as TLR7 and/or TLR9. Activation of these receptors in pDCs results in production of type I IFNs *via* the MyD88-dependent activation of nuclear factor-κB (NF-κB) and interferon regulatory factor 7 (IRF7) (7, 8) to induce the expression of IFN genes (9). Given the crucial function of IFNs in innate and adaptive immunity, IFN production must be tightly controlled to avoid detrimental and undesirable inflammatory responses. Indeed, many regulatory mechanisms have been described to modulate IFN responses at multiple levels (10). Among them, activation of NF-κB and IRFs has different contribution to IFN-α and IFN-λ gene expression through directly binding to their promoters (11, 12). In addition, recent studies suggest that activation of PI3K-Akt-mTOR pathway is critically required for TLR-mediated type I IFN production in human pDCs (13). Although previous studies suggest that type I and III IFN genes are regulated by a common mechanism in mouse embryonic fibroblasts, whether their expression in immune cells, particularly in their major producer pDCs, is regulated by a similar mechanism remains unclear.

MicroRNAs (miRs) are small, noncoding RNAs that regulate gene expression primarily at the posttranscriptional level. Emerging evidence supports a regulatory role for miRs in multiple immune processes (14). For example, miR-146a has been shown to regulate survival and maturation of human pDCs (15). In addition, direct roles of miRs in innate immune response have been shown in several studies (16). Of those, miR-146a, miR-155, and miR-21 induced by TLR activation serve as negative feedback regulators in inflammatory responses (17). Although the roles of miRs in inflammatory responses have been extensively studied, regulation of IFNs expression by miRs has not been widely appreciated. Yet recent studies strongly suggested that certain miRs play critical roles in regulation of type I IFN production in immune cells such as macrophages (18) and human pDCs (19).

MicroRNA-21 is an abundantly expressed miR in multiple types of mammalian cells including immune cells. Multiple validated targets of miR-21 have been identified, among which tumor suppressor phosphatase and tensin homolog (PTEN) and programmed cell death 4 (PDCD4) are the best described (20). Previous studies showed that miR-21 negatively regulated TLR-induced pro-inflammatory cytokine expression by targeting PDCD4 in macrophages (21, 22). However, whether miR-21 is also involved in regulation of TLR-mediated IFNs production remains unclear. Importantly, there is no evidence so far to show whether the recently described type III IFN can be regulated by certain miRs. In the present study, we demonstrated that the expression of miR-21 increased markedly in pDCs in response to TLR stimulation and miR-21 was required for optimal production of IFN-α and IFN-λ by pDCs. As a consequence, miR-21 expression enhanced host antiviral immune responses. Mechanistic studies provided further evidence showing that miR-21 promoted production of type I and III IFNs through targeting PI3K-Akt-mTOR signaling pathway. Overall, our study identifies miR-21 as a novel positive regulator of type I and III IFN in pDCs and suggests that miR-21 is crucial for modulation of pDCs activation and for promoting host antiviral immunity.

## Materials and Methods

### Mice

C57BL/6J mice were purchased from Beijing Vital River Laboratory Animal Technology Co., Ltd. *MiR-21^−/−^* mice with C57BL/6J background were purchased from Jackson Laboratory. Mice were maintained in a specific pathogen-free condition. Littermates with genotypes of *miR-21*^+/+^ and *miR-21^−/−^* mice were used for experiments. Experiments on mice were performed at 8–12 weeks of age with gender-matched littermates. All animal protocols were reviewed and approved by the Institute Animal Care and Use Committee of Tsinghua University.

### Construction of Bone Marrow Chimeras

CD45.1 of C57BL/6J background recipient mice were lethally irradiated by X-ray (4.5 Gy ×2) and then intravenously transferred with 2 × 10^6^ bone marrow leukocytes from the indicated donor mice. Chimeras were used for experiments 4 weeks after the initial reconstitution.

### Cell Isolation, Flow Cytometry Analysis, and Cell Sorting

For DC isolation, spleens or thymuses were cut and digested with collagenase III and Dnase I, followed by light-density separation and then immune-magnetic bead depletion using the procedure described elsewhere (23). For generation of Fms-like tyrosine kinase 3 (Flt3) ligand (FL)-cultured bone marrow-derived pDCs (24, 25) *in vitro*, total bone marrow cells were cultured for 7–9 days in culture medium consisting of RPMI1640 medium supplemented with essential and nonessential amino acids, 5.5 × 10^−5^mol/L 2-mercaptoethanol, 100 U/mL penicillin, 100 µg/mL streptomycin, and 10% FBS (Gibco) with 100 ng/mL recombinant murine FL (Peprotech). Dead cells were excluded by 7-AAD viability staining (eBioscience). DCs were stained with combinations of mAbs to CD11c (N418), SiglecH (eBio440C), B220 (RA3-6B2), CD172a (P84), CD8a (53-6.7), and CD24 (M1/69). CD11c^int^SiglecH^+^B220^+^ were used to gate pDCs population. The antibodies were purchased from eBioscience (San Diego, CA, USA) or Biolegend (San Diego, CA, USA). Cells were analyzed with a LSRII flow cytometer (BD) or sorted with a FACSAria III machine (BD). FACS data were analyzed and displayed by FlowJo software (Tree Star).

### DC Stimulation

Sorted pDCs were stimulated *in vitro* with TLR9 agonist CpG (1 μM ODN2216, InvivoGen) or TLR7 agonist R848 (1 µg/mL, InvivoGen). The recombinant cytokines mouse IL-3 (20 ng/mL) and mouse GM-CSF (20 ng/mL) were added as previously described (26). Cell viability was confirmed by 7-AAD and Annexin V assay (eBioscience). For inhibitor treatment, pDCs were pretreated with rapamycin, LY294002 inhibitors for 3 h or 4E1Rcat inhibitor (Selleck) for 6 h and were washed out before subsequent stimulation.

### Enzyme-Linked Immunosorbent Assay (ELISA)

Cytokines production was quantified by ELISA kits following the instructions of manufacturers. ELISA antibody sets for mouse IFN-α, IFN-β, IFN-λ, IL-6, and TNF-α were purchased from PBL Biomedical Laboratories.

### Immunoblotting Analysis

Immunoblots were performed as previously described (27). Briefly, cells were lysed with RIPA Lysis and Extraction Buffer (Pierce) containing protease inhibitor, phenylmethanesulfonyl fluoride, and phosphatase inhibitor (Pierce) on ice. Primary antibodies against phosphorylated mTOR (Ser2448), Akt; phosphorylated Akt (Thr308), phosphorylated 4E-binding protein 1 (4E-BP1) (Ser65), phosphorylated p70 ribosomal S6 protein (p70S6K) (Thr389), NF-κBp65, phosphorylated NF-κBp65 (Ser536), IκBα, PTEN, and PDCD4 are all from Cell Signaling Technology; antibody against IRF7 was from Santa Cruz Biotechnology. Anti-β-actin was purchased from Sigma.

### Reverse Transcription and Quantitative Real-time PCR (qPCR)

Total RNA was extracted from whole cell lysates by Trizol reagent (Invitrogen) and was reversely transcribed to cDNA with a PrimeScript RT Reagent Kit (Takara). qPCR was performed in triplicate samples using ABI 7900 thermal cycler. Threshold cycle numbers were normalized to triplicate samples amplified with primers specific for glyceraldehyde-3-phosphate dehydrogenase (*Gapdh*) or *U6*. Primer sequences are listed as following: *Ifna* sense: ARSYTGTSTGATGCARCAGGT, antisense: GGWACACAGTGATCCTGTGG; *Ifnl* sense: AGCTGCAGGTCCAAGAGCG, antisense: GGTGGTCAGGGCTGAGTCATT; *Irf7* sense: CAGCGAGTGCTGTTTGGAGAC, antisense: AAGTTCGTACACCTTATGCGG; *Gapdh* sense: ACCACAGTCCATGCCATCAC, antisense: TCCACCACCCTGTTGCTGTA; *miR-21*sense: CTCAACTGGTGTCGTGGAGTCGGCAATTCAGTTGAGTCAACATC, antisense: ACACTCCAGCTGGGTAGCTTATCAGACTGA; URP: TGGTGTCGTGGAGTCG; and U6 sense: CTCGCTTCGGCAGCACA, antisense: AACGCTTCACGAATTTGCGT.

### Construction of PTEN Expression and PTEN Knockdown Plasmids

The full-length murine PTEN cDNA was cloned into pMIG-modified vector (Addgene). PTEN shRNA was cloned into pLVshRNA-eGFP-Puro plasmid. PTEN shRNA forward: GATCCGCAGCTAAAGGAAGTGAATCTTTCAAGAGAAGATTCACTTCCTTTAGCTGCTTTTTTG, reverse: AATTCAAAAAAGCAGCTAAAGGAAGTGAATCTTCTCTTGAAAGATTCACTTCCTTTAGCTGCG.

### Lentiviral and Retroviral Transduction

For lentivirus package, 10 µg of PTEN shRNA plasmids or empty vectors were mixed with packaging plasmids (at ratio of 7:3:1) and were transfected into HEK293T cells by Lipofectamine 2000 (Invitrogen). 48 h posttransfection, virus-containing supernatants were collected and passed through a 0.45-µm filter (Millex). For lentiviral or retroviral transduction, 1 × 10^6^ FL-pDCs were resuspended with 1 mL virus-containing medium in 24-well plates precoated with RetroNectin (Takara) and subjected to spin infection. Cells were rested for 4 h at 37°C and then replated in fresh medium. After 24 h, cells were harvested for the subsequent experiments.

### Dual-Luciferase Reporter Assay

A PTEN 3′UTR luciferase reporter plasmid was generated by inserting full-length murine PTEN 3′UTR into the Xho I and Not I sites in the psiCHECK2 vector (Promega) downstream from the renilla luciferase coding sequence. The PTEN 3′UTR was PCR amplified from mouse genomic DNA with following primers: forward, 5′-CCGCTCGAGTCTTGTGCTGTGCAGCAG-3′ and reverse, 5′-AAGGAAAAAAGCGGCCGCGTGATGGCATTCACTGAACC-3′. Mutations within the putative miR-21 binding sites were generated by the following primers: forward, 5′-AACCGCGGTAAGAGAAATAAGCACCGTTTTCCAAG-3′ and reverse, 5′-AAAACGGTGCTTATTTCTCTTACCGCGGTTCAGATGTCTGAAGAT-3′. The FL-pDCs were plated in 24-well plates at 5 × 10^5^ cells per well. Transfection was performed in triplicate with Lipofectamine 2000 and 400 ng of the plasmids mixture (360 ng of the miR-21 mimic plasmid and 40 ng of the reporter vector). Luciferase assays for both firefly and renilla luciferase were performed 48 h after transfection with a Dual-Glo Luciferase Assay Kit (Promega). Luminescence was quantified using a Tecan SpectraFluor Plus Microplate Reader (Tecan). The renilla luciferase readings were normalized to the firefly luciferase activities in the corresponding well.

### Immunofluorescence and Confocal Microscopy

Immunofluorescence to detect IRF7 expression was performed as previously described (13, 28). Briefly, 2 × 10^5^ purified pDCs were cultured in 96-well round-bottom plates with 200 µL medium and were stimulated with 1 µM CpG ODN2216 for 3 h. Cells were stained with anti-mouse MHC II-FITC (Clone M5/114.15.2, eBioscience) and subsequently fixed with 2% paraformaldehyde and then permeabilized with 100% ice-cold methanol for 10 min at −20°C. A primary ployclonal antibody for mouse IRF7 (Santa Cruz) and an anti-rabbit IgG secondary antibody conjugated with Alexa Flour 647 (eBioscience) were used to detect IRF7 expression. 4 × 10^3^ cultured cells were seeded on a glass slide by cytospin and mounted using ProLong Antifade with DAPI (Invitrogen). Images were acquired using a confocal microscope (LSM 780; Zeiss, Inc.) and a 63× oil objective. Images were acquired sequentially using separate laser excitation to avoid any cross-talk between the fluorophore signals.

### Virus Infection

For *in vitro* herpes simplex virus 1 (HSV-1) infection, purified pDCs were infected with HSV-1 (wild-type F strain) at a dose of 2.5 × 10^6^ pfu/mL for 1 h. Cells were then washed with PBS and cultured in modified mouse osmolarity RPMI 1640 medium (DC medium). IFN-λ production was analyzed 18 h later. For *in vivo* studies, age- and gender-matched groups of mice received tail vein injection of HSV-1 (5 × 10^6^ pfu/mouse) in 200 µL of volume.

### Statistic Analysis

Statistical analysis was carried out using GraphPad Prism 5.0. Comparisons among multiple groups were calculated with the Student’s *t*-test or ANOVA. *P* values less than 0.05 were considered statistically significant.

## Results

### miR-21 Is Required for Robust Production of IFN-λ and IFN-α by pDCs

**Figure 1:**
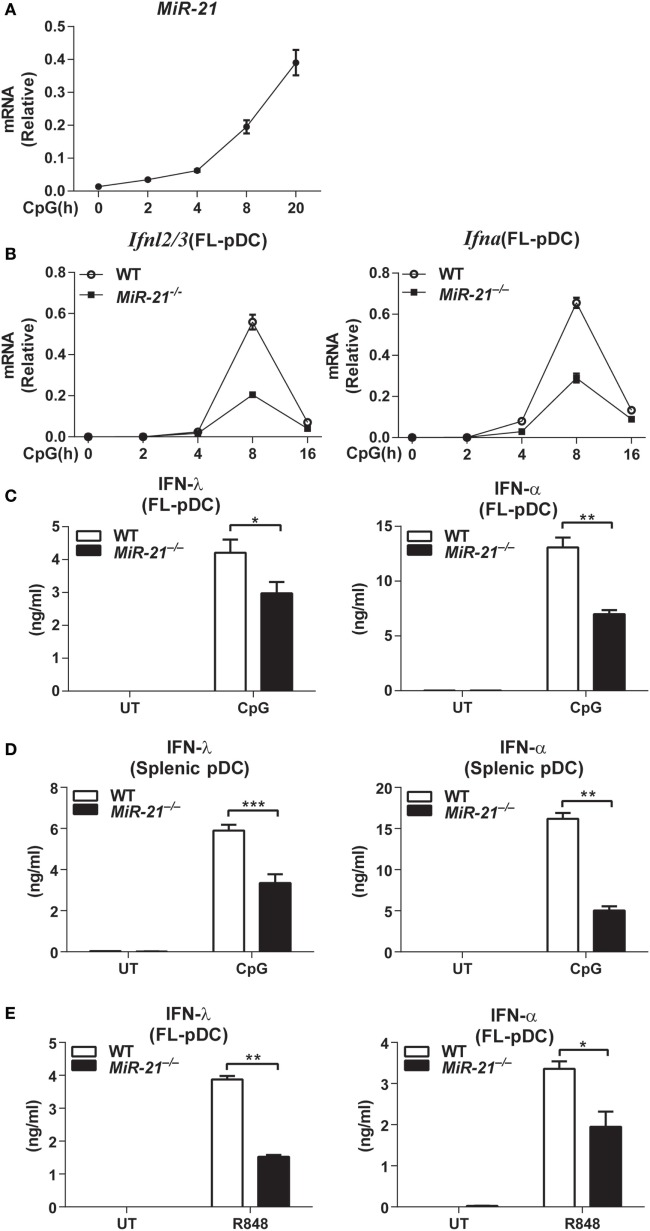
MicroRNA (miR)-21 selectively promotes the toll-like receptor-mediated interferons (IFNs) production by plasmacytoid dendritic cells (pDCs). **(A)** Quantitative real-time PCR (qPCR) analysis of miR-21 mRNA induced by CpG ODN (1 µM) in wild-type Fms-like tyrosine kinase 3 ligand (FL)-pDCs and was normalized to the expression of U6 in each sample. Data shown are from three independent experiments (mean ± SEM of biological triplicate determinants) **(B)**. qPCR analysis of *Ifnl2/3* and *Ifna* mRNA in wild-type and miR-21-deficient FL-pDCs stimulated with CpG ODN (1 µM) at indicated period. Data shown are from three independent experiments (mean ± SEM of biological triplicate determinants). **(C–E)** Enzyme-linked immunosorbent assay of IFN-λ and IFN-α production in FL-pDCs **(C)** or splenic pDCs **(D)** from wild-type and miR-21-deficient mice stimulated with CpG (1 µM) **(C,D)** or R848 (1 µg/mL) **(E)** for 20 h. Data shown are from three independent experiments (mean ± SEM of biological triplicate determinants) **(C–E)**. *P < 0.05 (unpaired Student’s *t*-test).

MicroRNA-21 expression is inducible by multiple stimuli in many types of mammalian cells (20). We found that deficiency of miR-21 does not affect development of bone marrow, splenic, or thymus DCs *in vivo* (Figures S1 and S2A–C in Supplementary Material) and differentiation of DCs *in vitro* (Figure S3A in Supplementary Material). These results prompted us to address whether miR-21 plays roles in regulation of DCs function. Interestingly, we found that miR-21 was strongly induced in FL-pDCs in response to CpG ODN stimulation (Figure 1A), indicating that high level of miR-21 may be correlated with pDCs activation. pDCs have been identified as potent secretors of IFN-α and IFN-λ, and the hallmark of pDCs activation is the rapid production of these IFNs upon recognition of viruses or non-self nucleic acids through TLR7 or TLR9 (29). To assess the role of miR-21 in pDC activation, FL-pDCs from wild-type and miR-21-deficient mice were stimulated with CpG ODN for different periods. qPCR analysis showed that expression of *Ifnl2/3* and *Ifna* was robustly induced in wild-type pDCs in response to CpG ODN stimulation, while expression of these two genes was much lower in miR-21-deficient pDCs (Figure 1B), suggesting that miR-21 is required for efficient induction of *Ifnl* and *Ifna* in pDCs. Consistently, protein levels of IFN-λ and IFN-α were also lower in the supernatants of miR-21-deficient pDCs than those of wild-type cells (Figures 1C,D). In contrast to high levels of IFN-λ and IFN-α induced by CpG stimulation, IFN-β is present at a negligible level in pDCs, and IFN-β expression did not differ significantly between wild-type and miR-21-deficient pDCs (Figure S3B in Supplementary Material). In addition, similar results were also observed in miR-21-deficient FL-pDCs when stimulated with a TLR7 agonist resiquimod (R848) (Figure 1E), indicating that defective production of IFN-λ and IFN-α by miR-21-deficient pDCs is not restricted to TLR9 activation. Previous study showed that XCR1^+^ cDCs are a subgroup of specialized cells to produce IFN-λ *in vivo* in response to poly(I:C) stimulation (26). However, we found that XCR1^+^ cDCs were unable to produce IFN-λ in response to CpG stimulation (Figure S3C in Supplementary Material), indicating that pDCs are the major subgroup of DCs to produce IFN-λ mediated by TLR9 activation.

Toll-like receptor stimulation can lead to apoptosis of pDCs, and it has been reported that miR-21 is involved in regulation of cell death (22). To rule out the possibility that the impaired production of IFN-λ and IFN-α was due to increased cell death of pDCs by miR-21 deficiency-induced apoptosis, we determined the apoptotic sensitivity of wild-type and miR-21-deficient pDCs. On CpG stimulation, wild-type and miR-21-deficient FL-pDCs showed comparable viability (Figure S4A in Supplementary Material), demonstrating that the impaired production of IFN-λ and IFN-α by miR-21-deficient pDCs is not due to the poor cell viability. In addition, we found that miR-21 deficiency did not alter the expression of *Tlr*9 in pDCs (Figure S4B in Supplementary Material), indicating that the defective production of these IFNs by miR-21-deficient pDCs is not due to a lower level of *Tlr9* expression. In contrast to the effects on IFN-α and IFN-λ production, lack of miR-21 did not affect expression of the prototypical pro-inflammatory cytokines IL-6 and TNF-α at mRNA and protein levels (Figure S5A in Supplementary Material). Taken together, these results suggest that miR-21 functions as a selective regulator of IFN production in pDCs.

### miR-21 Promotes IFN-λ and IFN-α Production by pDCs during Viral Infection

**Figure 2:**
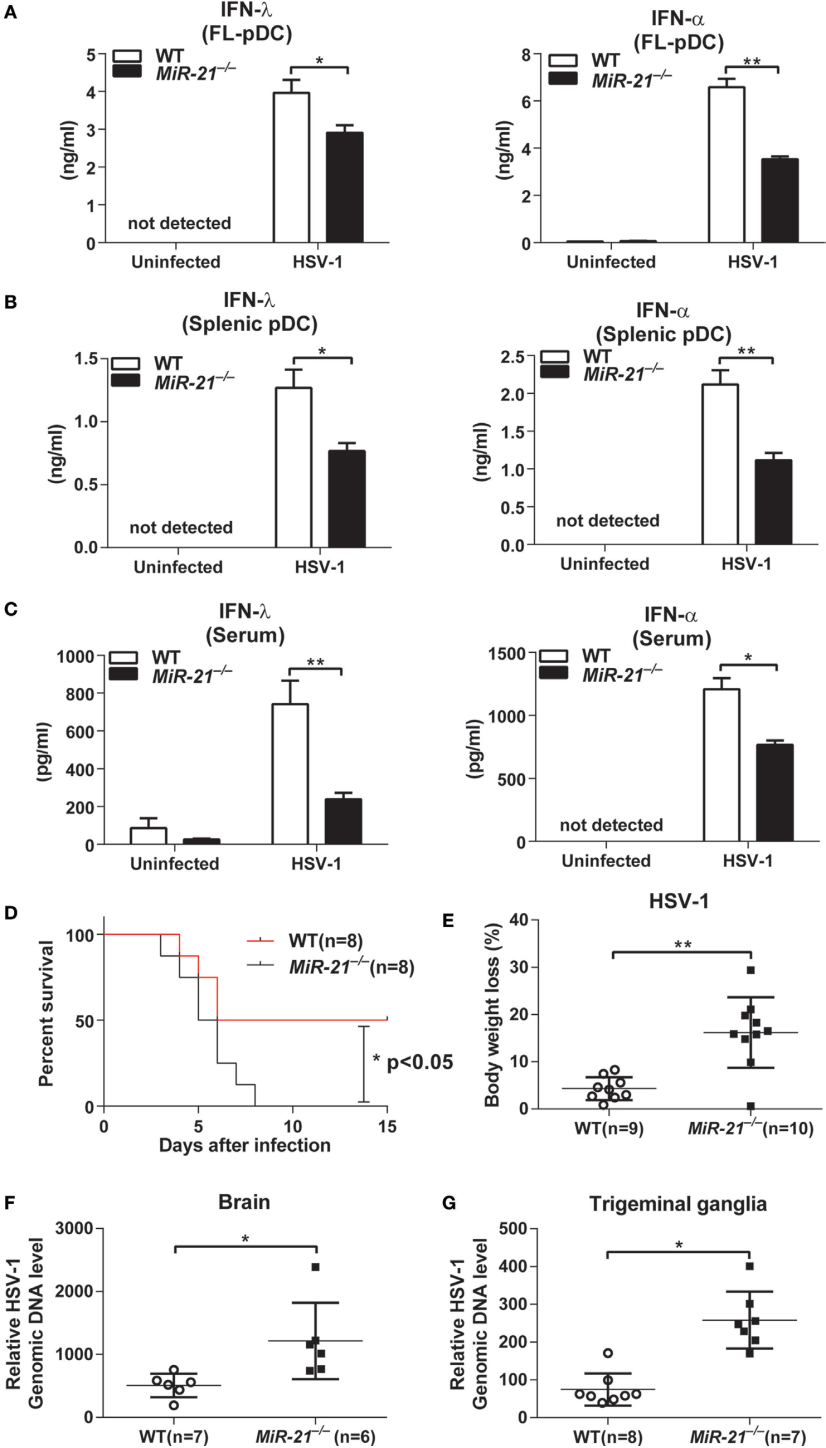
MicroRNA (miR)-21 suppresses herpes simplex virus 1 (HSV-1) propagation on viral infection. **(A,B)** Enzyme-linked immunosorbent assay (ELISA) of interferon (IFN)-λ or IFN-α in wild-type and miR-21-deficient Fms-like tyrosine kinase 3 ligand-plasmacytoid dendritic cells (FL-pDCs) **(A)** or splenic pDCs **(B)** infected with HSV-1 (2.5 × 10^6^ pfu/mL) for 18 h *in vitro*. **(C)** ELISA of IFN-λ and IFN-α in serum from wild-type and miR-21-deficient mice intravenously infected with HSV-1 (5 × 10^6^ pfu) for 7 h. **(D,E)** Survival rate **(D)** and body weight loss **(E)** of wild-type and miR-21-deficient mice infected with HSV-1 at various times. Viruses (1 × 10^7^ pfu) were given *via* tail vein injection. Body weight loss was recorded on day 4 postinfection. **(F)** Quantitative real-time PCR (qPCR) analysis of HSV-1 genomic DNA levels in the brains of wild-type and miR-21-deficient mice infected with HSV-1 (6 × 10^6^ pfu) *via* tail vein injection. Brain tissues were harvested on day 3 after infection. **(G)** qPCR analysis of HSV-1 genomic DNA levels in trigeminal ganglia (TG) of wild-type and miR-21-deficient mice infected with HSV-1 (6 × 10^6^ pfu/eye) *via* corneal infection. TG were isolated from mice on day 3 postinfection. Data shown are mean ± SEM and pooled from two independent experiments [**(A–D)**, *n* = 8 in each group; **(E–G)**, 6–10 mice in each group, and *n* value was indicated in the graph], *P < 0.05, **P < 0.01(unpaired Student’s *t*-test).

Having established that miR-21 deficiency impaired IFN-λ and IFN-α production induced by TLR ligand stimulation *in vitro*, we next sought to investigate whether miR-21 deficiency affects IFN production during viral infection. Therefore, FL-pDCs or splenic pDCs from wild-type and miR-21-deficient mice were infected with HSV-1, a dsDNA virus that can be recognized by TLR9 to induce type I and III IFN production. Consistent with our observation with CpG stimulation, we found that miR-21 deficiency markedly impaired production of IFN-λ and IFN-α in response to HSV-1 infection in both FL-pDCs and splenic pDCs (Figures 2A,B). Similarly, serum IFN-λ and IFN-α levels in miR-21-deficient mice were also lower than those in wild-type mice infected with HSV-1 (Figure 2C). Together, these results demonstrate that miR-21 deficiency impairs IFN-λ and IFN-α production during viral infection both *in vitro* and *in vivo*.

Both type I and III IFNs are the key mediators of host defense against viral infection. To evaluate the effects of miR-21 on viral infection *in vivo*, wild-type and miR-21-deficient mice were intravenously infected with a sublethal dose of HSV-1. We found that miR-21 deficiency exacerbated HSV-1-induced death of mice (Figure 2D), which suggests that miR-21 contributes to host defense against viral infection. In line with these observations, miR-21-deficient mice exhibited more severe body weight loss compared to that of wild-type mice (Figure 2E). In addition, levels of HSV-1 DNA in the brain of miR-21-deficient mice were higher than those of wild-type mice (Figure 2F). Similarly, viral loads in the TG of miR-21-deficient mice were also higher than those in wild-type animals infected by HSV-1 *via* cornea (Figure 2G). miR-21 is expressed in many types of mammalian cells. To rule out the possibility that miR-21 deficiency in non-hematopoietic cells may impair IFNs production *in vivo*, we generated chimeric mice by transferring bone marrow cells from wild-type and miR-21-deficient mice to C57BL/6 mice. On HSV-1 infection, miR-21-deficient chimeras exhibited lower serum levels of IFN-α and IFN-λ compared to those of wild-type chimeras (Figure S5B in Supplementary Material), indicating that deficiency of miR-21 in hematopoietic cells contributes to impaired induction of IFNs *in vivo*. Collectively, these data demonstrate that miR-21 contributes to host defense against viral infection likely *via* regulating the production of type I and III IFNs by pDCs.

### miR-21 Promotes IRF7 Expression and Nuclear Translocation in pDCs

**Figure 3:**
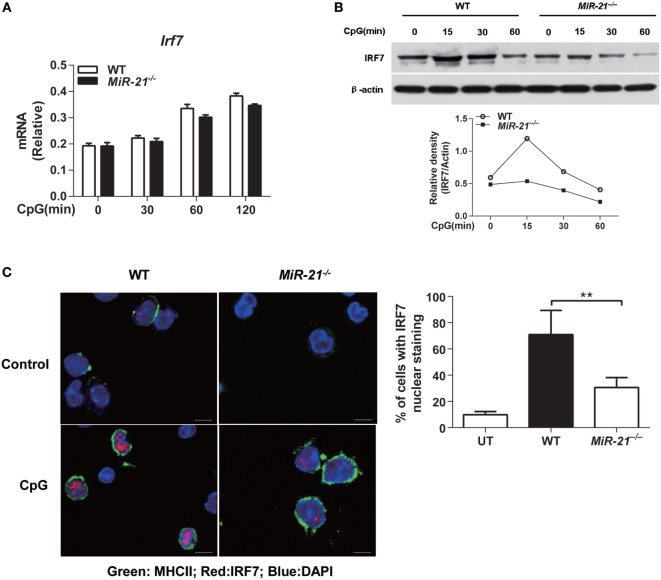
MicroRNA (miR)-21 is required for interferon regulatory factor 7 (IRF7) translation and nuclear translocation in toll-like receptor-activated plasmacytoid dendritic cells (pDCs). **(A,B)** Quantitative real-time PCR analysis of *Irf7* mRNA **(A)** or immunoblot analysis of IRF7 protein [**(B)**, upper] from wild-type and miR-21-deficient Fms-like tyrosine kinase 3 ligand-pDCs (FL-pDCs) stimulated with CpG ODN (1 µM) for the indicated periods. Relative density of band from each time point was shown as gray level ratio (IRF7 versus β-actin). β-Actin served as a loading control. Data shown are representative of three **(A)** or two **(B)** independent experiments [mean ± SEM of biological triplicate determinants in **(A)**]. **(C)** Immunofluorescence of nuclear translocation of IRF7 (Alexa Flour 647, red) in sorted pDCs left untreated (UT) or stimulated with CpG ODN (1 µM) for 2.5 h (left). Cells were visualized using the membrane staining of MHC II (FITC), and the nucleus was identified using DAPI. Data shown are representative cells from three independent experiments. Bar, 5 µM. (Right) Quantitative analysis of pDCs with IRF7 nuclear translocation as at left. At least 50–70 cells from 5 donors were analyzed. Nuclear translocation was considered positive when at least 20% of the IRF7 fluorescence was localized in the nucleus. Data are shown as mean ± SEM of FL-pDCs from four mice each group and are representative of three independent experiments. **P < 0.01 (one-way ANOVA with *post hoc* Bonferroni *t*-test).

It is well established that IRF7 is the master transcription factor of IFN-α and IFN-λ expression in mouse pDCs (28). To investigate the mechanisms by which miR-21 modulates the expression of these IFNs, expression and nuclear translocation of IRF7 were examined. We found that miR-21 deficiency did not affect *Irf7* mRNA expression by pDCs on CpG stimulation (Figure 3A), but expression of IRF7 protein was significantly lower than that of wild-type pDCs after CpG stimulation. The subsequent nuclear translocation of IRF7 was also reduced in miR-21 null cells (Figures 3B,C), suggesting that miR-21 regulates expression of IFN-α and IFN-λ through modulating IRF7 expression at posttranscriptional level in pDCs. It is worth noting that CpG-induced IRF7 protein expression has a very short half-life in pDCs (Figure 3B), which is consistent with a previous study (30). It is known that TLR-induced activation of NF-κB contributes to type I and III IFN production by pDCs. We found that phosphorylation of p65 and IκBα exhibited a similar pattern between wild-type and miR-21-deficient pDCs in response to CpG stimulation (Figure S6A in Supplementary Material), suggesting that miR-21 deficiency does not affect canonical NF-κB activation in pDCs. Taken together, these data reveal that miR-21 promotes expression of IFN-α and IFN-λ *via* modulating transcription factor IRF7 without altering activation of canonical NF-κB signaling.

### miR-21 Facilitates Activation of PI3K-Akt-mTOR Signaling to Regulate IRF7 Protein Synthesis

**Figure 4:**
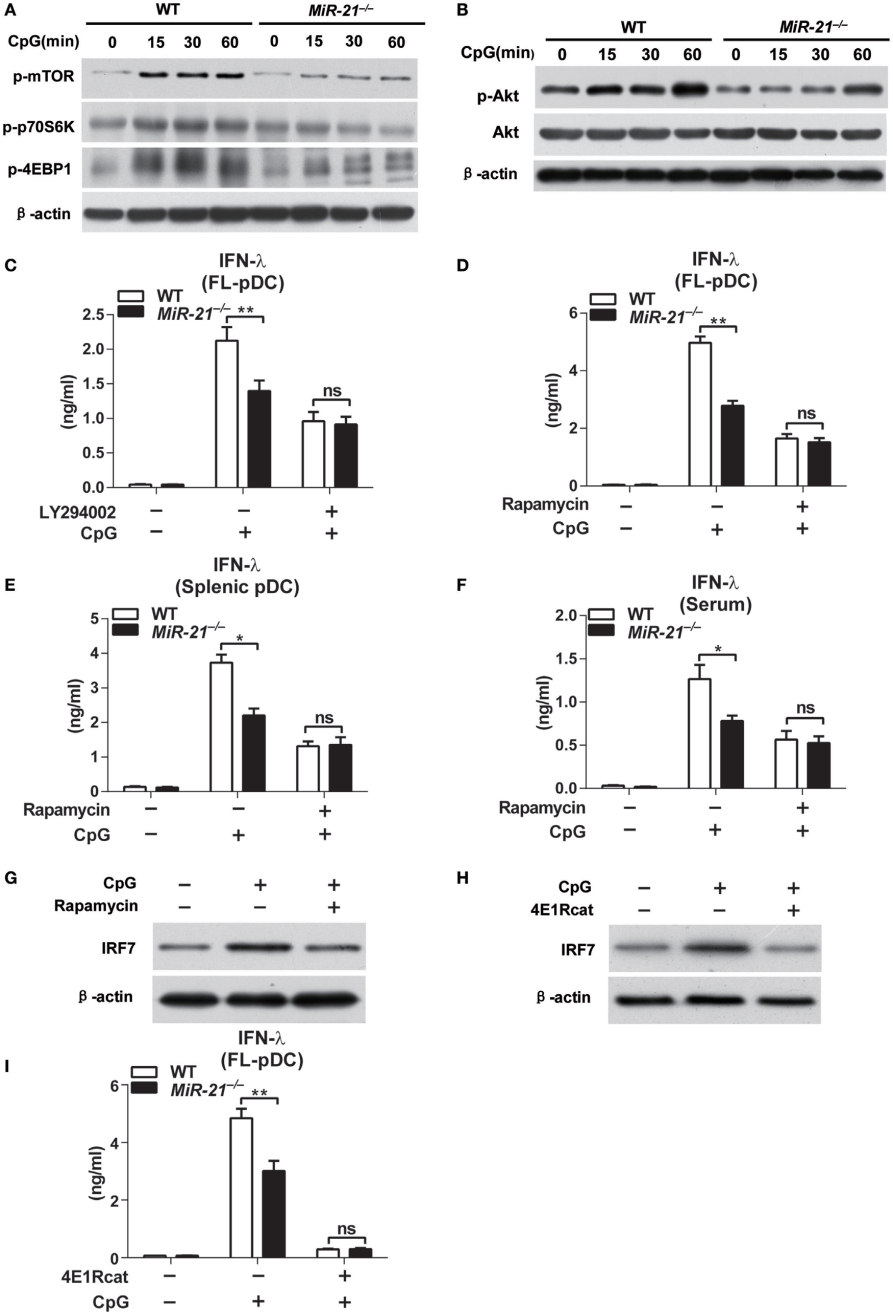
MicroRNA (miR)-21 deficiency impairs induction of interferon (IFN)-λ in plasmacytoid dendritic cells (pDCs) *via* suppression of PI3K-Akt-mTOR pathway. **(A,B)** Immunoblot analysis of mTOR, p70s6k, and 4E-binding protein 1 (4E-BP1) phosphorylation **(A)** and Akt phosphorylation **(B)** in wild-type and miR-21-deficient Fms-like tyrosine kinase 3 ligand-pDCs (FL-pDCs) stimulated with CpG ODN (1 µM) for the indicated periods. β-Actin and total Akt served as loading controls. **(C–E)** Inhibitory effects of LY294002 (500 nM) **(C)** and rapamycin (300 nM) **(D)** on CpG ODN (1 μM)-induced IFN-λ production in FL-pDCs **(C,D)** or splenic pDCs **(E)** from wild-type and miR-21-deficient mice. **(F)** Inhibitory effect of rapamycin on serum IFN-λ production of wild-type and miR-21-deficient mice injected with CpG ODN-coated with cationic liposomes for 3 days (*n* = 4 in each group). **(G,H)** Immunoblot analysis of IRF7 in wild-type FL-pDCs pretreated with 300 nM rapamycin or DMSO for 3 h following stimulation with CpG ODN (1 µM) for 15 min **(G)** or pretreated with 50 µM 4E1Rcat (4E1R) or DMSO 6 h following stimulation with CpG ODN (1 µM) for 15 min **(H)**. **(I)** Enzyme-linked immunosorbent assay of IFN-λ in wild-type and miR-21-deficient FL-pDCs pretreated with 4E1R (50 µM) or DMSO for 8 h and subsequently stimulated with CpG ODN (1 µM) for 12 h. Data shown are mean ± SEM from three independent experiments **(C–E,I)** or pooled from two independent experiments [**(F)**, *n* = 8]. *P < 0.05, **P < 0.01 (two-way ANOVA with *post hoc* Bonferroni *t*-test).

Recent studies suggest that TLR-triggered PI3K-Akt-mTOR signaling plays an essential role in type I IFN induction by pDCs (31). IRF7 protein synthesis is dependent on activation of mTOR and phosphorylation of mTOR downstream mediators. p70S6K phosphorylation enhances protein translation, while phosphorylation of eukaryotic initiation factor 4E-BP1 releases 4E-BP1 from eIF4E and facilitates translation. Hence, mTOR signaling is a key regulator of IRF7 expression in pDCs. To determine whether miR-21 regulates IRF7 expression through targeting mTOR pathway, we assessed TLR-induced mTOR activation by detecting its phosphorylation form in wild-type and miR-21-deficient pDCs. On CpG stimulation, mTOR phosphorylation was strongly induced within 15 min and sustained for at least 1 h in wild-type pDCs, while phosphorylation of mTOR in miR-21-deficient pDCs showed only a minimal increase (Figure 4A), suggesting that miR-21 is necessary for sufficient activation of mTOR. Consistent with the pattern of mTOR activation, phosphorylation of p70S6K and 4E-BP1 induced by CpG stimulation was also decreased in miR-21-deficient pDCs (Figure 4A). Given that mTOR, p70S6K, and 4E-BP1 are all downstream targets of the kinase Akt (32), we next tested whether miR-21 deficiency also affected TLR-induced Akt activation. The results showed that deficiency of miR-21 significantly suppressed TLR-mediated phosphorylation of Akt without altering expression of total Akt (Figure 4B), indicating that miR-21 promotes mTOR activation through enhancing activation of the upstream kinase Akt in pDCs. Together, these data suggest that miR-21 is a critical positive regulator that promotes PI3K-Akt-mTOR signaling cascades during pDCs activation, which contributes to the maintenance of IRF7 protein expression.

The role of PI3K-Akt-mTOR signaling in regulation of type I IFN in pDCs has been reported (31). However, whether the production of type III IFN is also regulated *via* this signaling pathway remains unknown. Therefore, we determined whether inhibition of PI3K-Akt-mTOR signaling by chemical inhibitors LY294002 and rapamycin affected IFN-λ production in pDCs. We found that pharmacological inhibition of both PI3K and mTOR significantly suppressed TLR-induced IFN-λ production in wild-type and miR-21-deficient pDCs (Figures 4C,D), suggesting that PI3K-Akt-mTOR activation is crucial for robust induction of IFN-λ in pDCs. Notably, CpG induced similar levels of IFN-λ in wild-type and miR-21-deficient pDCs after pretreatment of PI3K inhibitor LY294002 or mTOR inhibitor rapamycin (Figures 4C–E), demonstrating that miR-21 promotes IFN-λ production by acting on PI3K-Akt-mTOR signaling. In addition, rapamycin pretreatment resulted in comparable levels of serum IFN-λ in wild-type and miR-21-deficient mice in response to CpG stimulation *in vivo* (Figure 4F). These results demonstrate that miR-21 selectively promotes TLR-induced IFN-α and IFN-λ production by pDCs through regulating PI3K-Akt-mTOR activation both *in vitro* and *in vivo*.

Given that mTOR regulates IRF7 expression and IFN production through its downstream translational repressor 4E-BP1 in pDCs (33), we thus examined effects of rapamycin on IRF7 expression. The results showed that rapamycin treatment decreased the protein levels of IRF7 (Figure 4G), indicating that mTOR activation is required for enhancement of IRF7 synthesis in pDCs. Moreover, inhibition of 4E-BP1, a key factor that regulates protein expression at the level of translation initiation *via* promoting assembly of the eIF4E–eIF4G complex (34), also suppressed IRF7 protein expression (Figure 4H), confirming that mTOR targets IRF7 expression *via* direct regulation of 4E-BP1. As a result, IFN-λ production mediated by CpG stimulation was decreased in 4E1Rcat-treated pDCs (Figure 4I). We also confirmed that, at the concentration we used, all the inhibitors did not apparently affect the survival of pDCs (Figure S7 in Supplementary Material). Collectively, these data provide evidence that miR-21 modulates IFN-α and IFN-λ responses in pDCs through targeting PI3K-Akt-mTOR signaling to regulate IRF7 expression at translational level.

### miR-21 Suppresses PTEN to Promote Akt Phosphorylation in pDCs

**Figure 5:**
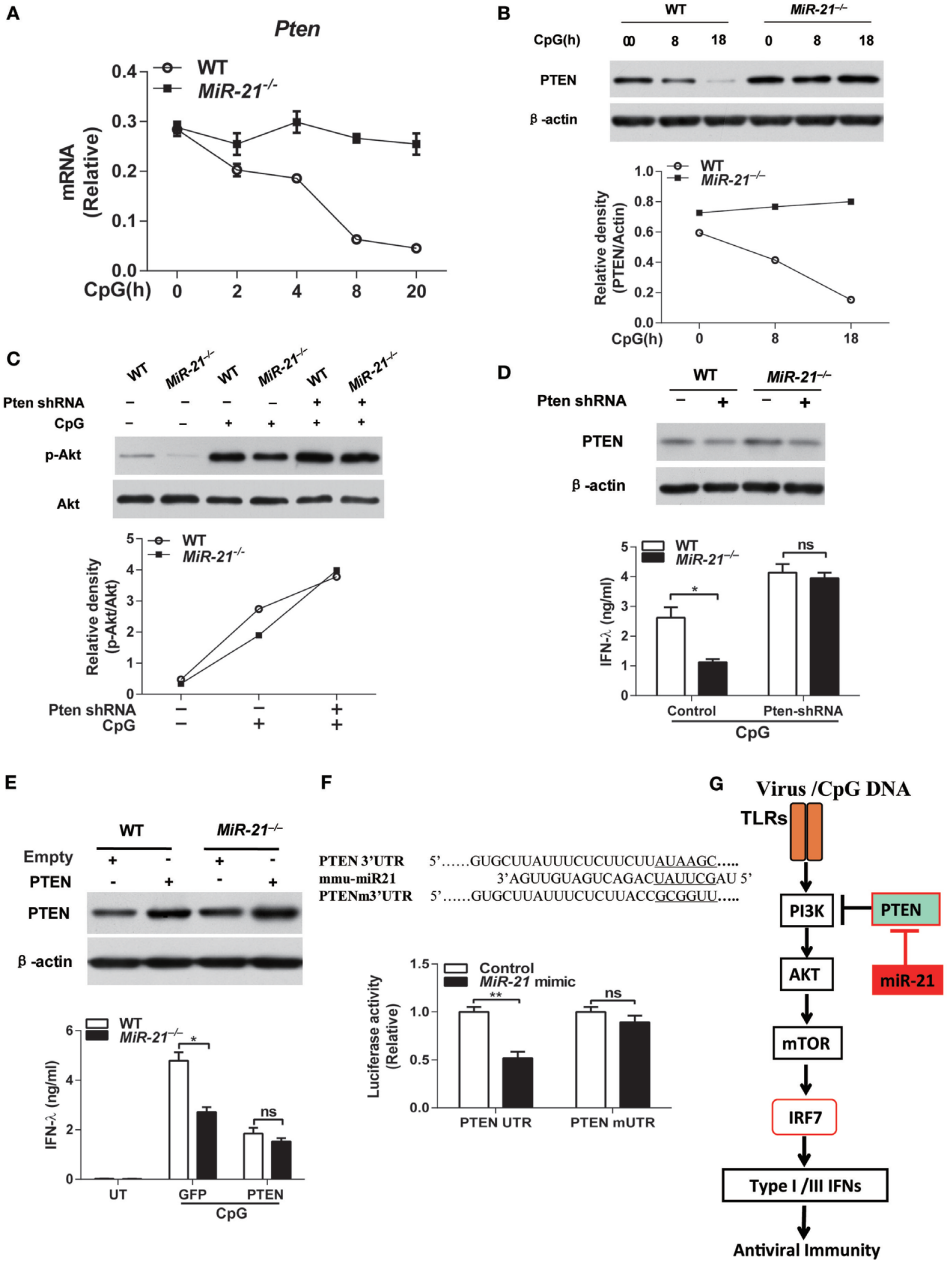
MicroRNA (miR)-21 regulates toll-like receptor (TLR)-induced interferon (IFN) responses through targeting phosphatase and tensin homolog (PTEN). **(A)** Quantitative real-time PCR analysis of *Pten* mRNA in wild-type and miR-21-deficient Fms-like tyrosine kinase 3 ligand-plasmacytoid dendritic cells (FL-pDCs) stimulated with CpG ODN (1 µM) for the indicated periods. Data are representative of two independent experiments (mean ± SEM of biological triplicate determinants). **(B)** Immunoblot analysis of PTEN expression in wild-type and miR-21-deficient FL-pDCs treated with CpG ODN (1 µM) for the indicated periods [**(B)**, upper panel] or Akt phosphorylation in wild-type and miR-21-deficient FL-pDCs transduced with lentiviral particles expressing PTEN-specific shRNA or nontargeting control shRNA and subsequently stimulated with CpG ODN (1 µM) for 30 min [**(C)**, upper panel]. Relative density of PTEN [**(B)**, lower panel] or p-Akt [**(C)**, lower panel] was accordingly normalized to expression of β-actin or total Akt. **(D,E)** Enzyme-linked immunosorbent assay of IFN-λ in wild-type and miR-21-deficient FL-pDCs transduced with lentiviral particles with PTEN-specific shRNA or nontargeting control shRNA **(D)** or transduced with retroviral particles expression GFP or murine PTEN **(E)** and subsequently stimulated with CpG ODN (1 µM) for 20 h. PTEN protein expression was confirmed by immunoblotting accordingly. Data shown are mean ± SEM of three independent experiments using the pool of eight mice per experiment. *P < 0.05 (unpaired Student’s *t*-test). **(F)** A nucleotide comparison between the miR-21 seed sequence and the PTEN 3′UTR sequence and PTEN 3′UTR mutant sequence (the six nucleotides in the miR-21 seed region were underlined) and relative renilla luciferase activities in FL-pDCs co-transfected with a PTEN 3′UTR psiCHECK plasmid (psiCHECK2-PTEN-UTR) or a PTEN 3′UTR mutant plasmid (psiCHECK2-PTEN-mUTR) and miR-21 mimic or control mimic. Data from one representative experiment of two performed is shown as mean ± SEM of biological triplicate determinants. **P < 0.01 (unpaired Student’s *t*-test). **(G)** A model of miR-21 in regulation of IFN responses in pDCs. TLR-induced type I and III IFNs production is dependent on interferon regulatory factor 7 (IRF7) activation in pDCs. TLR stimulation also activates PI3K-Akt-mTOR signaling pathway that is required for sufficient IFN production in pDCs. In miR-21-deficient cells, miR-21 deficiency suppresses phosphorylation of Akt through promoting expression of PTEN, a direct target of miR-21 that functions as a negative regulator of Akt activation, thereby inhibits activation of mTOR and subsequent IRF7 expression and nuclear translocation to reduce IFNs production by pDCs.

Having identified that miR-21 regulates TLR-mediated IFN-λ production through modulating PI3K-Akt-mTOR pathway, we next sought to investigate the direct target by which miR-21 regulates PI3K-Akt-mTOR signaling. It has been well established that PTEN negatively regulates Akt activation in various types of cells including immune cells (35–37). Interestingly, PTEN has been identified as one of the direct targets of miR-21 (38). We thus tested whether miR-21 regulated Akt activation in pDCs through inhibition of PTEN expression. CpG stimulation led to decreased *Pten* expression in wild-type pDCs that is inversely correlated with CpG-mediated induction of miR-21 expression (Figure 1A), but did not affect the PTEN mRNA level in miR-21-deficient pDCs (Figure 5A), indicating that miR-21 may directly regulate *Pten* gene expression in pDCs. Moreover, PTEN protein level was higher both at basal level and in CpG-stimulated condition in miR-21-deficient pDCs than that in wild-type pDCs (Figure 5B), suggesting that the lack of miR-21 increases PTEN expression. In addition, knockdown of PTEN expression by transduction of lentiviral shRNA particles into pDCs promoted Akt phosphorylation induced by CpG stimulation (Figure 5C). These data demonstrate that miR-21 facilitates Akt activation by inhibiting expression of PTEN, one canonical target of miR-21. Interestingly, PTEN knockdown in miR-21-deficient pDCs indeed restored TLR9-induced IFN-λ production to a similar level compared to that in wild-type cells (Figure 5D), suggesting that PTEN is the key upstream molecule that suppresses IFN-λ response in miR-21-deficient pDCs. Conversely, PTEN overexpression suppressed IFN-λ production by pDCs in response to CpG stimulation (Figure 5E). Collectively, these data suggest that PTEN functions as a negative regulator of TLR-mediated IFN-λ production and is responsible for impaired IFN-λ expression in miR-21-deficient pDCs. It has been shown that PTEN is a validated target of miR-21 in other cell types (39). To investigate whether PTEN is also a direct target of miR-21 in pDCs, we performed luciferase assay by co-transfection of miR-21 mimic with a PTEN 3′UTR or PTEN 3′UTR mutant reporter construct in FL-pDCs. miR-21 mimic markedly decreased PTEN 3′UTR-driven luciferase activities in pDCs, but did not affect mutant PTEN 3′UTR-induced luciferase activities (Figure 5F), confirming that PTEN is also a validated target of miR-21 in pDCs (Figure 5G).

## Discussion

Type I IFN (IFN-α) and type III IFN (IFN-λ) are key mediators in the establishment of a multifaceted antiviral response of host. Given the critical role of type I and III IFNs in innate and adaptive immunity, IFN responses must be under tight control to prevent pathogenic responses (40). pDCs are highly specialized cells that can produce large amounts of IFN-α and IFN-λ in response to TLR agonists or viral infections. However, the molecular mechanisms that regulate this important antiviral response are still not completely understood. Many regulatory mechanisms involving surface receptors, intracellular factors and exogenous factors, and virus-encoded molecules have been shown to modulate IFNs responses of pDCs (41, 42). In this study, we identified miR-21 as a key modulator required for TLR9-induced robust production of IFN-α and IFN-λ by pDCs. Interestingly, miR-21 deficiency did not affect TLR-induced activation of canonical NF-κB signaling, a key transcription factor responsible for IFNs expression in pDCs. Instead, deficiency of miR-21 resulted in upregulation of its target PTEN, which suppressed phosphorylation of Akt, leading to defective activation of mTOR and its downstream targets p70S6K and 4E-BP1, thereby decreased the expression of IRF7 at post transcriptional level. Therefore, our study demonstrate that miR-21 regulates IRF7 expression and subsequent type I and III IFNs production *via* targeting PI(3)K-Akt-mTOR pathway in pDCs. It is worth noting that evidence for regulation of IFN-λ expression still remains scarce. Hence, our findings provide the first evidence that IFN-λ production can be regulated by miRs.

The expression of miR-21 can be significantly induced by various stimuli in many cell types. For example, studies have shown that elevation of miR-21 could orchestrate PI(3)K-Akt-mTOR signaling *via* targeting PTEN in various cell types including human colorectal cancer cells (43), bladder cancer cells (44), and glomerular mesangial cells (45). In addition, induction of miR-21 in macrophages negatively regulated production of pro-inflammatory cytokines through targeting PTEN (22). Interestingly, we found that miR-21 was also highly induced in pDCs in response to TLR stimulation, and such induction might be an indicator of activation of pDCs. However, the exact functional outcome of such increase of miR-21 in pDCs is unclear. Here, we demonstrated that miR-21 positively regulated TLR-mediated production of IFN-α and IFN-λ by pDCs through targeting PTEN and mTOR signaling. These findings suggest that induction of miR-21 appears to exert different effects in different cell types. As an example, miR-21 promotes TLR9-induced Akt phosphorylation and the subsequent expression of type I and III IFNs by targeting PTEN in pDCs, whereas it negatively regulates TLR4-induced IL-10 expression through targeting PDCD4 in macrophages. Given that each miR may target multiple genes, it is therefore not surprising that the same miR regulates distinct responses in different cell types (46). In line with this view, miR-21 induction by TLR9 did not affect PDCD4 expression in pDCs (Figure S6B in Supplementary Material), another established target of miR-21, which was previously identified as a regulator of type I IFN in macrophages (47).

The tumor suppressor gene PTEN is a validated target of miR-21, which displays an inverse correlation with miR-21 expression at the protein level in cancer cells (43). Recently, several lines of evidence have supported an essential role of PTEN in regulating innate immune responses (39). For example, one recent study demonstrates a pivotal function of PTEN in antiviral immunity (37). In MEF cells, PTEN controls the nuclear translocation of interferon regulatory factor 3 (IRF3), a master transcription factor responsible for IFN production, into the nucleus by dephosphorylating the negative phosphorylation site (Ser97) of IRF3 *via* the phosphatase activity of PTEN. Therefore, PTEN functions as a positive regulator of IFN-β in MEF cells. Notably, such regulation of PTEN is independent of the PI(3)K-Akt pathway. In contrast, we found PTEN, as the target of miR-21, negatively regulated TLR-mediated production of IFN-α and IFN-λ in pDCs. The different effects of PTEN observed are not unexpected as PTEN regulates the expression of IFN-α and IFN-λ through targeting PI(3)K-Akt pathway in pDCs, whereas regulation of IFN-β production by PTEN in MEF cells is independent of PI(3)K and Akt. Furthermore, it is well known that IRF7, but not IRF3, is the major master transcription factor responsible for type I IFN production in pDCs (7, 30, 48, 49). Hence, deficiency of PTEN further enhances IRF7-mediated expression of IFN, which is consistent with our observation that induction of miR-21 by TLR stimulation decreased PTEN expression in pDCs. Thus, our findings further support that PTEN is critical in regulation of type I and III IFNs production and host defense against viral infection, which is most likely through multiple mechanisms.

The role of miRs in regulating type I IFN production has attracted much attention given its importance in the cross-talk between host and virus (16). Indeed, expression of several miRs induced by infection of certain viruses may participate in antiviral responses (50). Here, we show that miR-21 is crucial for sufficient IFN-α and IFN-λ production in antiviral immune responses both *in vitro* and *in vivo*. Although miR-21 is expressed in many types of cells, we have verified that miR-21 exerts its antiviral effects primarily in immune cells using the bone marrow transfer approach. Previous studies suggest that pDCs are the primary source of IFN-α after iv inoculation of viruses including HSV-1 (51, 52). Therefore, the impaired production of IFN-α and IFN-λ caused by miR-21 deficiency *in vivo* is more likely due to defective pDCs function.

In summary, our findings demonstrate that miR-21 functions as a positive regulator for optimal production of type I and III IFNs by pDCs and provide clear evidence that the PI(3)K-Akt-mTOR pathway is involved in the regulation of IFN-λ production in pDCs. Interestingly, miR-21 expression can also be induced by type I IFNs, indicating that an amplification loop for induction of miR-21 takes place during viral infection (53). Hence, miR-21 may serve as a potential target for modulating antiviral immune responses.

## Ethics Statement

All animal protocols were reviewed and approved by the Institute Animal Care and Use Committee of Tsinghua University.

## Author Contributions

FL performed experiments and prepared the data; CL performed some experiments; XH provided valuable suggestion and revised the manuscript; YS supervised experiments and wrote the manuscript; and LW designed and supervised the project and wrote the manuscript.

## Conflict of Interest Statement

The authors declare that the research was conducted in the absence of any commercial or financial relationships that could be construed as a potential conflict of interest.

## Supplementary Material

Figure S1 Surface markers on plasmacytoid dendritic cells (pDCs) in wild-type and microRNA (miR)-21-deficient mice. Cells were stained with the indicated combinations of surface markers and were analyzed by flow cytometry. Analysis is representative of two independent experiments (*n* = 6).

Figure S2 Flow cytometry analysis of cell population of bone marrow dendritic cells (DCs) **(A)**, splenic DCs **(B)**, and thymus DCs **(C)** from wild-type and microRNA-21-deficient mice. Combinations of surface markers SiglecH, B220 and CD11c were used to gate plasmacytoid dendritic cells (pDCs) (B220^+^SiglecH^hi^CD11c^int^) and cDCs (SiglecH^low^CD11c^hi^), respectively. Subgroup of DCs were further gated with the indicated combinations of surface markers CD172a and CD24 (thymus DCs) or CD172a and CD8a (splenic DCs). Numbers in quadrants indicate percentage of pDCs or cDCs (left). (Right) Quantification of cells numbers of subgroup DCs as at left. Data shown are mean ± SEM and pooled from two independent experiments (*n* = 6), no significance (ns) (unpaired Student’s *t*-test).

Figure S3 **(A)** Flow cytometry analysis of cell population of bone marrow cultured with Fms-like tyrosine kinase 3 ligand-dendritic cell (FL-DCs). Data shown are mean ± SEM and pooled from two independent experiments (*n* = 6), no significance (ns) (unpaired Student’s *t*-test). **(B)** Enzyme-linked immunosorbent assay (ELISA) of interferon (IFN)-β production in FL-plasmacytoid dendritic cells (pDCs) from wild-type and microRNA (miR)-21-deficient mice stimulated with CpG (1 µM) for 20 h. **(C)** XCR1^+^ cDCs does not produce IFN-λ in response to CpG stimulation. ELISA of IFN-λ production in XCR1^+^ cDCs from wild-type and miR-21-deficient mice stimulated with CpG (1 µM) for 20 h. Data shown are mean ± SEM of one representative (*n* = 8) from two independent experiments. **P* < 0.05, ***P* < 0.01(unpaired Student’s *t*-test).

Figure S4 MicroRNA (miR-21) deficiency does not affect plasmacytoid dendritic cells (pDCs) viability and *Tlr9* transcript level in toll-like receptor (TLR)-activated pDCs. **(A)** Flow cytometry analysis of Fms-like tyrosine kinase 3 ligand (FL)-pDCs viability from wild-type and miR-21-deficient mice in the presence of CpG ODN (1 µM) for 16 h. PDCs were stained with Annexin V and 7-AAD. Numbers in quadrants were indicated percentage cells. Data shown are mean ± SEM of four mice each group from one experiment. **(B)** Quantitative real-time PCR analysis of *Tlr9* mRNA in wild-type and miR-21-deficient FL-pDCs stimulated with CpG ODN (1 µM) for indicated periods. Data are representative of two independent experiments (mean ± SEM of biological triplicates).

Figure S5 MicroRNA (miR)-21 deficiency does not affect canonical pro-inflammatory cytokines production in plasmacytoid dendritic cells (pDCs). **(A)** Enzyme-linked immunosorbent assay (ELISA) of interleukin (IL)-6 and tumor necrosis factor alpha (TNF-α) production (upper panel) or quantitative real time PCR analysis of *Il6* and *Tnf*α gene expression (lower panel) in Fms-like tyrosine kinase 3 ligand-pDCs from wild-type and miR-21-deficient mice stimulated with CpG (1 µM) for 20 h (upper panel) or at indicated periods (lower panel). Data shown are mean ± SEM of one representative from two independent experiments (upper, *n* = 4 mice per group, lower, mean ± SEM with biological triplicate determinants). **(B)** Hematopoietic cells are responsible for IFN-α and IFN-λ production to herpes simplex virus 1 (HSV-1) infection *in vivo*. ELISA of IFN-α (A) and IFN-λ (B) in serum from wild-type and miR-21-deficient chimeras infected with HSV-1 (5 × 10^6^ pfu) *via* iv for 7 h. Data shown are mean ± SEM pooled from two independent experiments (*n* = 8). ***P* < 0.01 (unpaired Student’s *t*-test).

Figure S6 Immunoblot analysis of nuclear factor-κB phosphorylation, IκBα expression **(A)**, and programmed cell death 4 (PDCD4) expression **(B)** in wild-type and miR-21-deficient Fms-like tyrosine kinase 3 ligand-plasmacytoid dendritic cells stimulated with CpG ODN (1 µM) for the indicated periods. β-Actin serves as a loading control. Data are representative of three independent experiments.

Figure S7 Apoptotic death of isolated mouse plasmacytoid dendritic cells pretreated with vehicle or 300 nM rapamycin (Rap), 500 nM LY294002 (LY), 50 µM 4E1Rcat (4E1R), assessed by staining with Annexin V and propidium iodide. Numbers in quadrants indicate percent cells in each. Data are representative of two independent experiments (*n* = 6).
