# Supplementary figures and images for: MicroRNA-21: A Positive Regulator for Optimal Production of Type I and Type III Interferon by Plasmacytoid Dendritic Cells
Source: Front Immunol. 2017 Aug 21;8:947. doi: 10.3389/fimmu.2017.00947 (PMC5567078; doi:10.3389/fimmu.2017.00947)

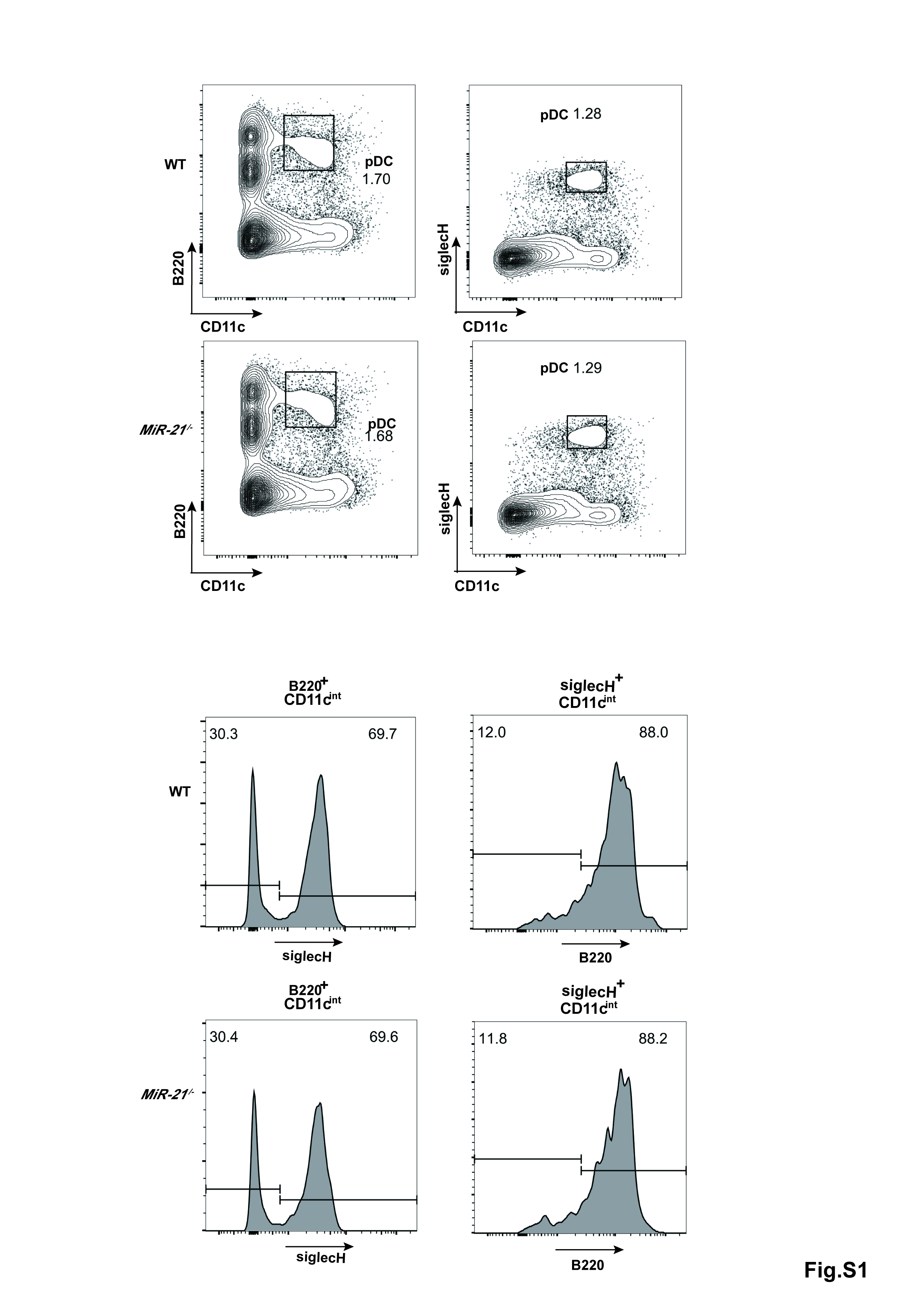

Supplement: Figure S1 — Surface markers on plasmacytoid dendritic cells (pDCs) in wild-type and microRNA (miR)-21-deficient mice. Cells were stained with the indicated combinations of surface markers and were analyzed by flow cytometry. Analysis is representative of two independent experiments (n = 6). [file Image_1.tif]

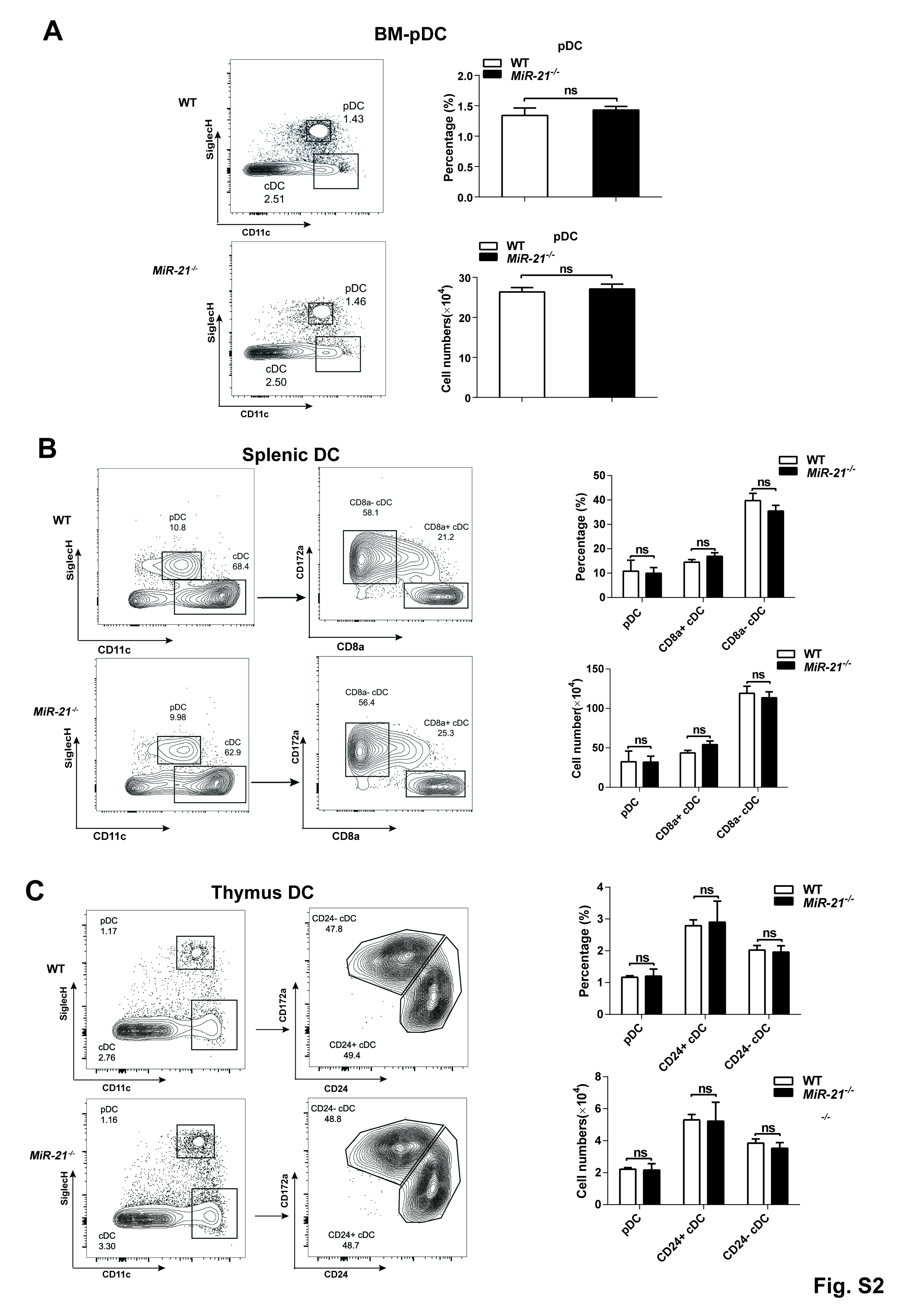

Supplement: Figure S2 — Flow cytometry analysis of cell population of bone marrow dendritic cells (DCs) (A), splenic DCs (B), and thymus DCs (C) from wild-type and microRNA-21-deficient mice. Combinations of surface markers SiglecH, B220 and CD11c were used to gate plasmacytoid dendritic cells (pDCs) (B220+SiglecHhiCD11cint) and cDCs (SiglecHlowCD11chi), respectively. Subgroup of DCs were further gated with the indicated combinations of surface markers CD172a and CD24 (thymus DCs) or CD172a and CD8a (splenic DCs). Numbers in quadrants indicate percentage of pDCs or cDCs (left). (Right) Quantification of cells numbers of subgroup DCs as at left. Data shown are mean ± SEM and pooled from two independent experiments (n = 6), no significance (ns) (unpaired Student’s t-test). [file Image_2.tif]

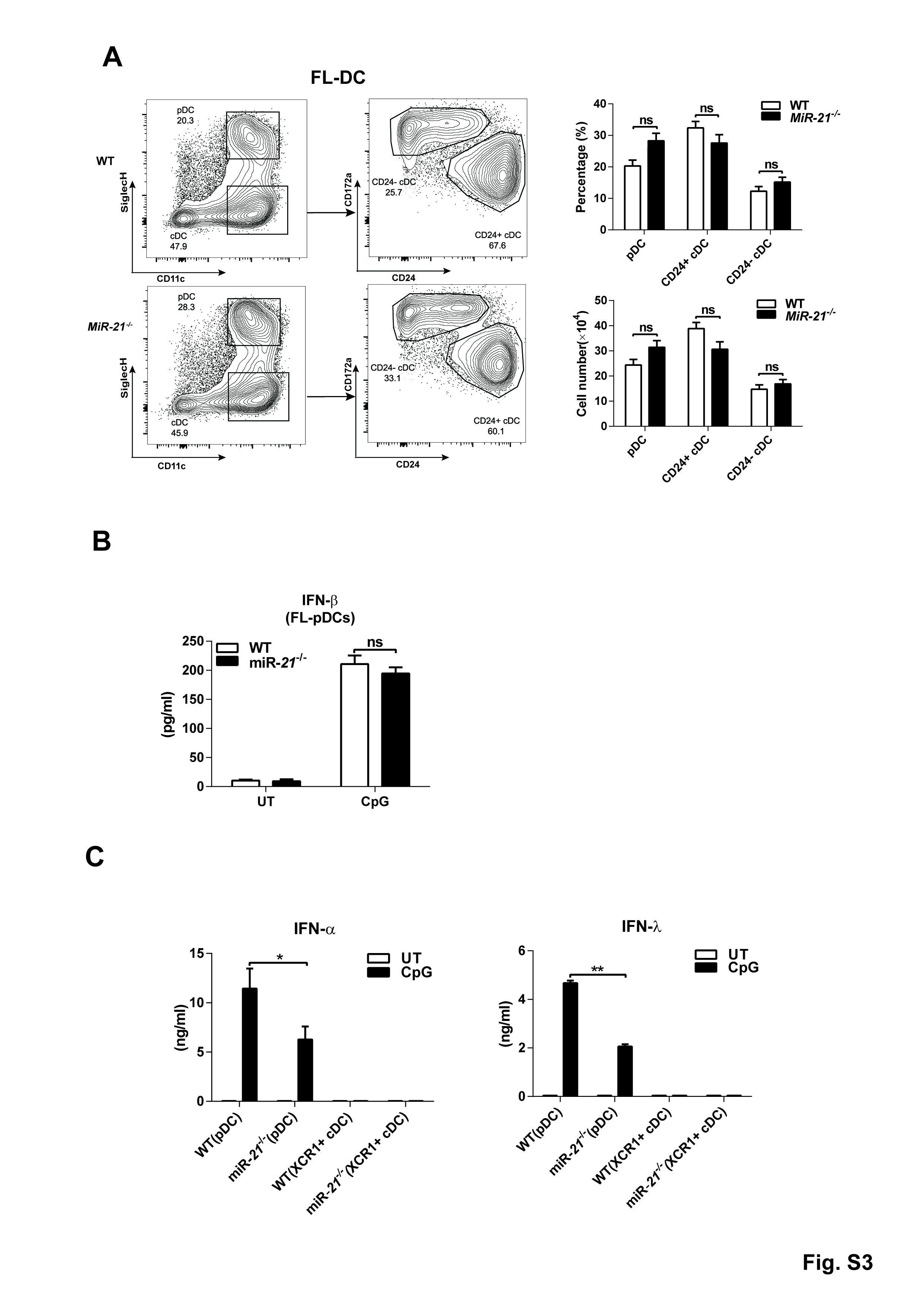

Supplement: Figure S3 — (A) Flow cytometry analysis of cell population of bone marrow cultured with Fms-like tyrosine kinase 3 ligand-dendritic cell (FL-DCs). Data shown are mean ± SEM and pooled from two independent experiments (n = 6), no significance (ns) (unpaired Student’s t-test). (B) Enzyme-linked immunosorbent assay (ELISA) of interferon (IFN)-β production in FL-plasmacytoid dendritic cells (pDCs) from wild-type and microRNA (miR)-21-deficient mice stimulated with CpG (1 µM) for 20 h. (C) XCR1+ cDCs does not produce IFN-λ in response to CpG stimulation. ELISA of IFN-λ production in XCR1+ cDCs from wild-type and miR-21-deficient mice stimulated with CpG (1 µM) for 20 h. Data shown are mean ± SEM of one representative (n = 8) from two independent experiments. *P < 0.05, **P < 0.01(unpaired Student’s t-test). [file Image_3.tif]

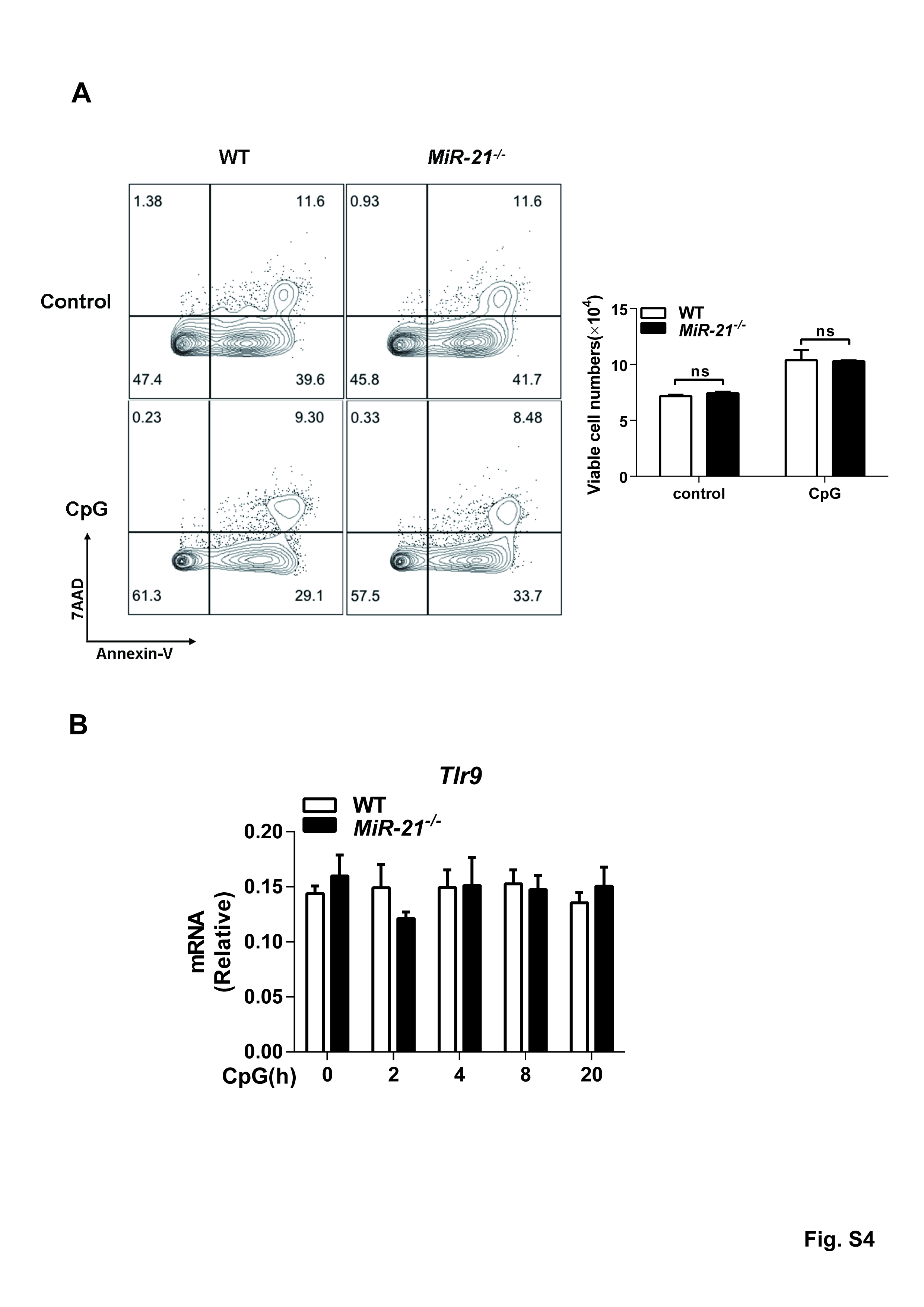

Supplement: Figure S4 — MicroRNA (miR-21) deficiency does not affect plasmacytoid dendritic cells (pDCs) viability and Tlr9 transcript level in toll-like receptor (TLR)-activated pDCs. (A) Flow cytometry analysis of Fms-like tyrosine kinase 3 ligand (FL)-pDCs viability from wild-type and miR-21-deficient mice in the presence of CpG ODN (1 µM) for 16 h. PDCs were stained with Annexin V and 7-AAD. Numbers in quadrants were indicated percentage cells. Data shown are mean ± SEM of four mice each group from one experiment. (B) Quantitative real-time PCR analysis of Tlr9 mRNA in wild-type and miR-21-deficient FL-pDCs stimulated with CpG ODN (1 µM) for indicated periods. Data are representative of two independent experiments (mean ± SEM of biological triplicates). [file Image_4.tif]

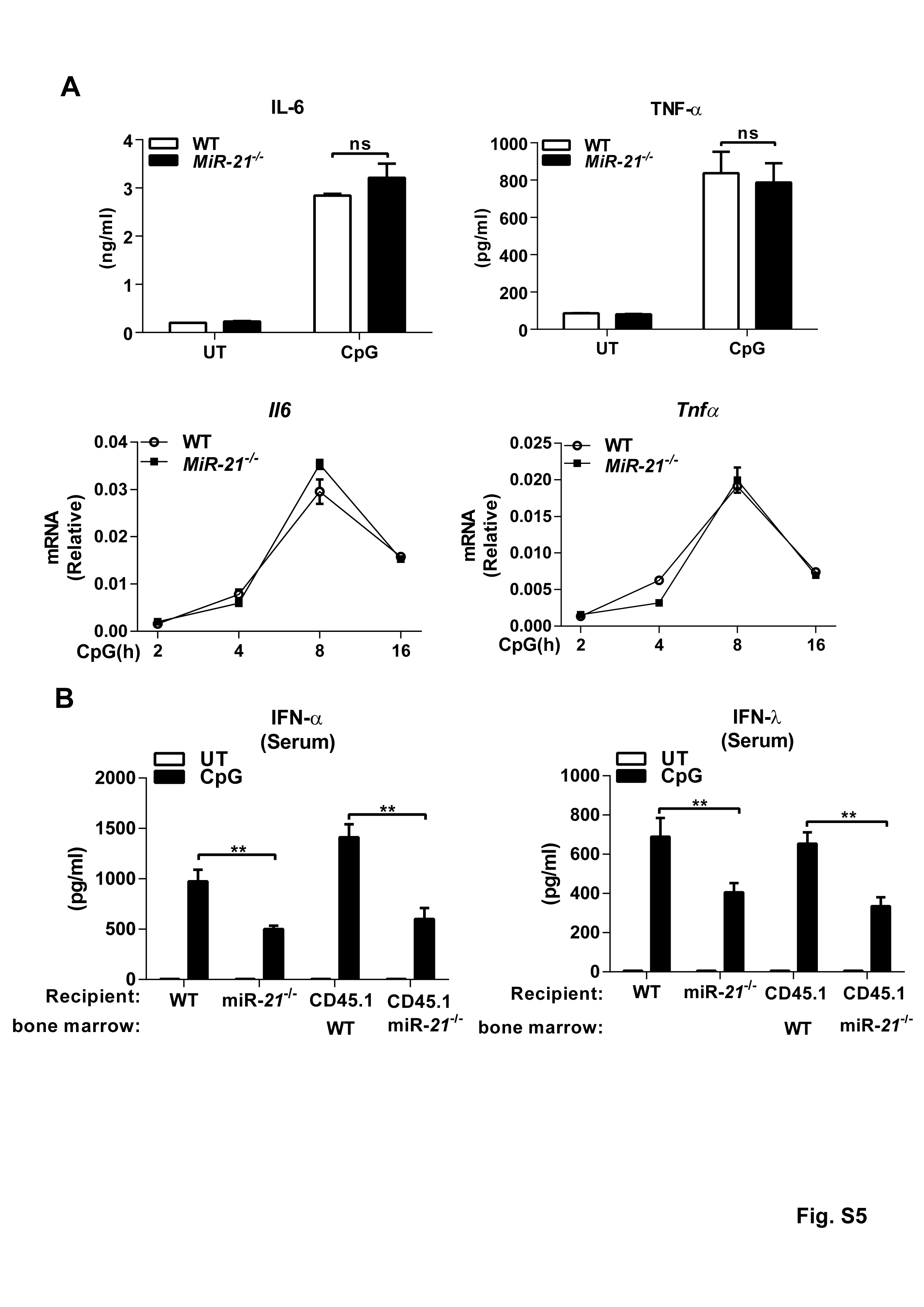

Supplement: Figure S5 — MicroRNA (miR)-21 deficiency does not affect canonical pro-inflammatory cytokines production in plasmacytoid dendritic cells (pDCs). (A) Enzyme-linked immunosorbent assay (ELISA) of interleukin (IL)-6 and tumor necrosis factor alpha (TNF-α) production (upper panel) or quantitative real time PCR analysis of Il6 and Tnfα gene expression (lower panel) in Fms-like tyrosine kinase 3 ligand-pDCs from wild-type and miR-21-deficient mice stimulated with CpG (1 µM) for 20 h (upper panel) or at indicated periods (lower panel). Data shown are mean ± SEM of one representative from two independent experiments (upper, n = 4 mice per group, lower, mean ± SEM with biological triplicate determinants). (B) Hematopoietic cells are responsible for IFN-α and IFN-λ production to herpes simplex virus 1 (HSV-1) infection in vivo. ELISA of IFN-α (A) and IFN-λ (B) in serum from wild-type and miR-21-deficient chimeras infected with HSV-1 (5 × 10^6^ pfu) via iv for 7 h. Data shown are mean ± SEM pooled from two independent experiments (n = 8). **P < 0.01 (unpaired Student’s t-test). [file Image_5.tif]

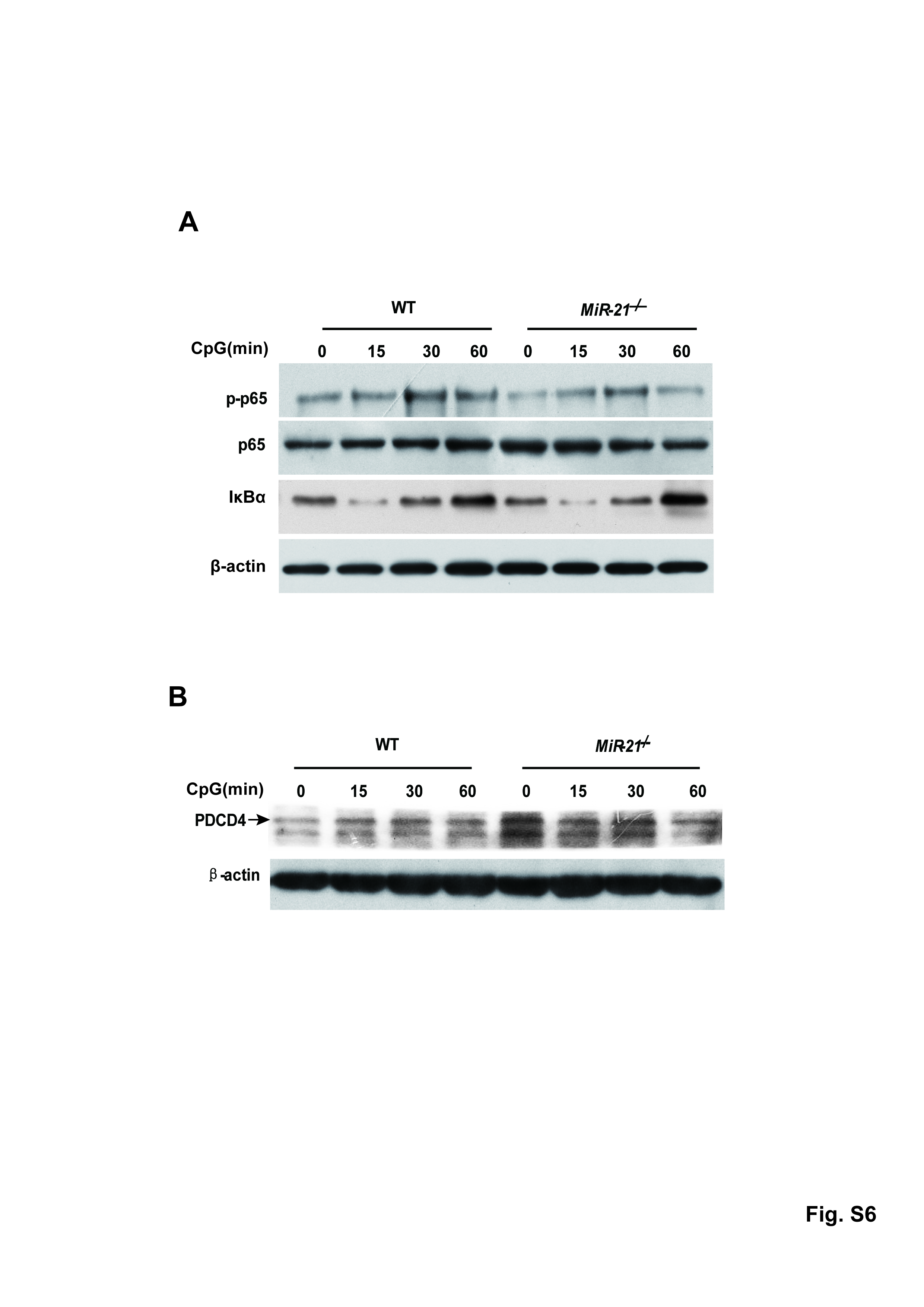

Supplement: Figure S6 — Immunoblot analysis of nuclear factor-κB phosphorylation, IκBα expression (A), and programmed cell death 4 (PDCD4) expression (B) in wild-type and miR-21-deficient Fms-like tyrosine kinase 3 ligand-plasmacytoid dendritic cells stimulated with CpG ODN (1 µM) for the indicated periods. β-Actin serves as a loading control. Data are representative of three independent experiments. [file Image_6.tif]

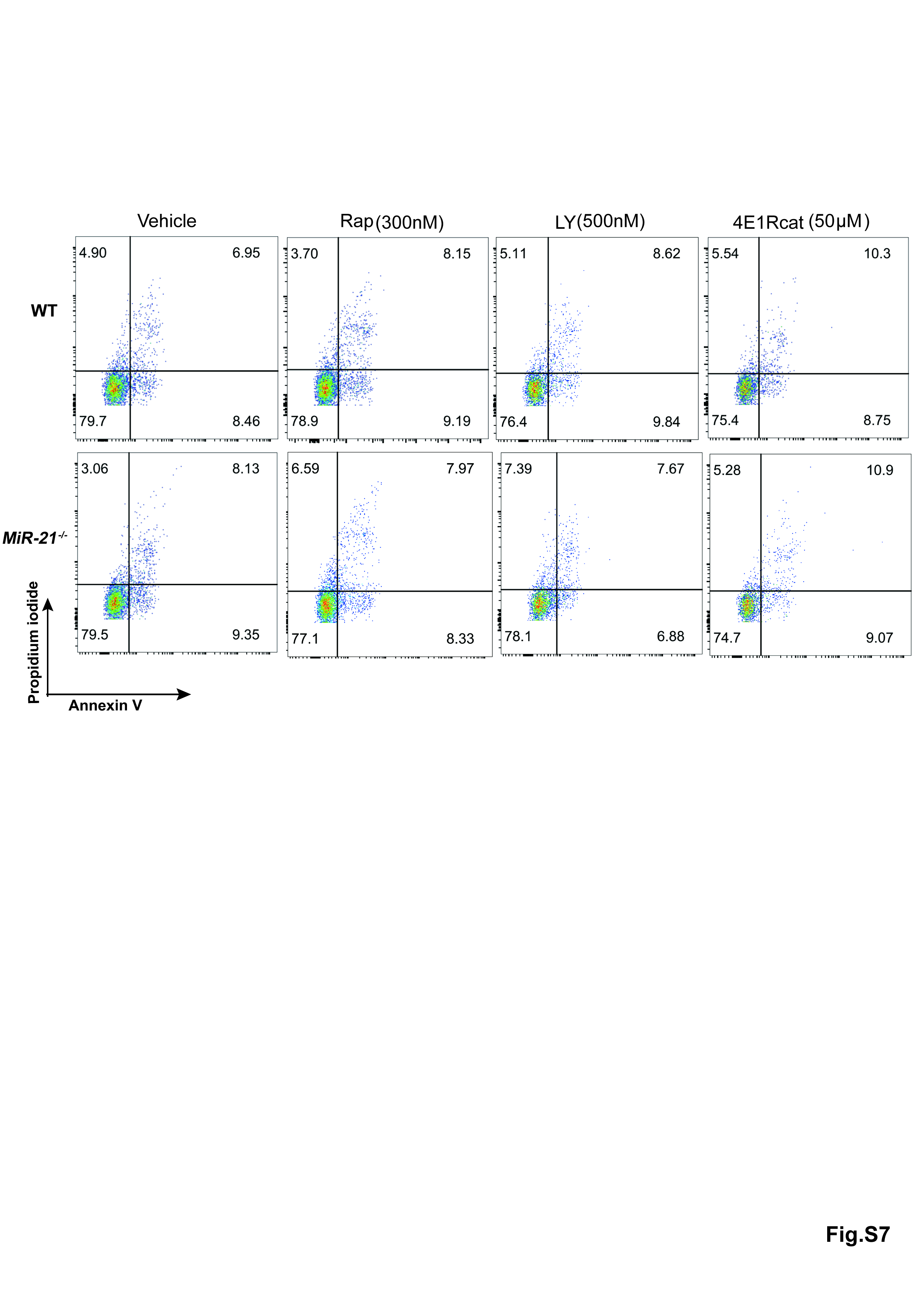

Supplement: Figure S7 — Apoptotic death of isolated mouse plasmacytoid dendritic cells pretreated with vehicle or 300 nM rapamycin (Rap), 500 nM LY294002 (LY), 50 µM 4E1Rcat (4E1R), assessed by staining with Annexin V and propidium iodide. Numbers in quadrants indicate percent cells in each. Data are representative of two independent experiments (n = 6). [file Image_7.tif]
